# Tsang’s resolution enhancement method for imaging with focused illumination

**DOI:** 10.1038/s41377-025-01791-4

**Published:** 2025-04-11

**Authors:** Alexander Duplinskiy, Jernej Frank, Kaden Bearne, A. I. Lvovsky

**Affiliations:** Department of Physics, University of Oxford, Oxford, OX1 3PU UK

**Keywords:** Imaging and sensing, Super-resolution microscopy

## Abstract

A widely tested approach to overcoming the diffraction limit in microscopy without disturbing the sample relies on substituting widefield sample illumination with a structured light beam. This gives rise to confocal, image scanning, and structured illumination microscopy methods. On the other hand, as shown recently by Tsang and others, subdiffractional resolution at the detection end of the microscope can be achieved by replacing the intensity measurement in the image plane with spatial mode demultiplexing. In this work, we study the combined action of Tsang’s method with image scanning. We experimentally demonstrate superior lateral resolution and enhanced image quality compared to either method alone. This result paves the way for integrating spatial demultiplexing into existing microscopes, contributing to further pushing the boundaries of optical resolution.

## Abstract (web summary)

Improving imaging resolution and quality by combining Tsang’s spatial mode demultiplexing with scanning a focused illumination beam over the sample.

## Graphical abstract

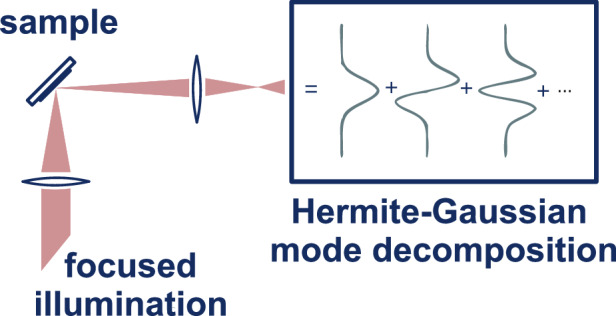

## Introduction

The ability to achieve resolution beyond the diffraction limit of light has revolutionized the field of microscopy. Superresolution techniques have enabled researchers to visualize structures and processes with unprecedented detail, leading to new discoveries and insights. The demand for better resolving optical systems has been traditionally driven by biological studies, where the delicate nature of samples often does not allow direct interaction. Therefore, non-invasive methods operating in the far-field have become highly popular as practical means to enhance resolution^1–5^.

Currently, many powerful superresolution methods exploit non-linear interactions between light and certain fluorescent samples. A prominent example is Stimulated Emission Depletion (STED) microscopy^6,7^, which uses an additional laser to selectively suppress fluorescence around the targeted focal spot, effectively reducing the point spread function beyond the conventional diffraction limit. Similarly, techniques like Photoactivated Localization Microscopy (PALM)^8,9^ and Stochastic Optical Reconstruction Microscopy (STORM)^10,11^ achieve super-resolved images through precise localization of individual fluorescent molecules activated in a sparse manner. The Separation by Lifetime Tuning (SPLIT) technique^12,13^ takes advantage of temporal dynamics in fluorescence, offering improved resolution for densely packed fluorescent signals and allows reducing the intensity required for depletion. These methods manage to achieve more than ten-fold resolution improvement and localize emitting particles with nanometer precision.

In contrast, linear superresolution techniques, although offering a more modest resolution enhancement, typically around a factor of two, have the advantage of not relying on any specific sample properties or active interaction with the sample. Methods such as confocal microscopy^14–16^, Image Scanning Microscopy (ISM)^17–22^, and Structured Illumination Microscopy (SIM)^4,23,24^ are not only universally compatible with all types of fluorescent dyes but can also be employed for coherent imaging of reflective samples. This versatility makes them highly adaptable tools suitable for a broad spectrum of imaging tasks and experimental conditions.

Confocal microscopy is one of the most established and widely implemented superresolution techniques that operate through non-uniform illumination^14–16^. In this method, the sample is scanned under a focused illumination beam while a pinhole placed in the image plane acts as a spatial filter, allowing only the light from the center of the illuminated object area to pass through. The intensity distribution recorded with a bucket detector with respect to the position of the scan forms a high-resolution image of the object.

Building upon this concept, image scanning microscopy (ISM)^17–19^ replaces the pinhole and the bucket detector with a detector array or a camera^17,18,20^. Snapshots recorded at each scanning step are combined into the final image using the pixel reassignment routine^21,22^. While providing the same lateral resolution, ISM eliminates the optical loss caused by the pinhole in the confocal setup.

An alternative linear far-field imaging resolution improvement method, proposed by Tsang et al.^25^, is based upon decomposing the incoming field in the detection plane into an orthonormal basis of transverse modes, such as Hermite–Gaussian (HG) modes, and measuring the amplitude or intensity of each mode. From these measurements, the original object can be reconstructed^26^. This method, which we refer to as Hermite–Gaussian Imaging (HGI), emerged from fundamental discoveries in quantum measurement theory in recent years^25–35^. This method allows one to not only achieve sub-Rayleigh precision but also, in some cases, reach the ultimate resolution limits allowed by quantum mechanics^25,29^.

In contrast to ISM, confocal microscopy, and other methods that achieve resolution enhancement by applying a transverse structure to the illuminating beam, HGI aims at improving the opposite end of the imaging system — the detection of light. Hence it is natural to ask whether both approaches can be combined with a cumulative effect. In this work, we implement this combination and demonstrate that applying HGI in the scanning paradigm results in resolution and image quality improvement compared to both techniques used independently.

Illumination- and detection-based linear superresolution methods have previously been combined in incoherent imaging using a confocal setup and Fresnel Incoherent Correlation Holography (FINCH)^36^. The resulting technique named CINCH (Confocal Incoherent Correlation Holography) did not demonstrate significant improvement compared to FINCH alone and performed at a level similar to SIM, however, avoiding sophisticated illumination patterns^37^. The main advantage gained by confocal illumination in this setup was the improvement of image quality by blocking out-of-focus light rather than further enhancing the lateral resolution.

## Results

### Image scanning for lateral resolution improvement

The effective point spread function (PSF) of an imaging system with a focused illumination beam can be expressed as a product of the illumination and detection PSFs: ${\rm{PS}}{{\rm{F}}}_{{\rm{ISM}}}={\rm{PS}}{{\rm{F}}}_{\det }\cdot {\rm{PS}}{{\rm{F}}}_{{\rm{il}}}$. Assuming that both PSFs are Gaussian with the widths *σ*_il_ and *σ*_det_, we have (for details see “Materials and methods” section):

1 $${\rm{PS}}{{\rm{F}}}_{{\rm{ISM}}}\left(x\right)\propto \exp \left(-\frac{{x}^{2}}{2{\sigma }_{\det }^{2}}\right)\exp \left(-\frac{{x}^{2}}{2{\sigma }_{{\rm{il}}}^{2}}\right)\propto \exp \left(-\frac{{x}^{2}}{2{\sigma }_{{\rm{ISM}}}^{2}}\right)$$

with

2 $${\sigma }_{{\rm{ISM}}}=\frac{{\sigma }_{{\rm{il}}}{\sigma }_{\det }}{\sqrt{{\sigma }_{{\rm{il}}}^{2}+{\sigma }_{\det }^{2}}}$$

Usually, the same objective lens is used both to focus the beam onto the specimen and to collect the light from it, meaning equal numerical apertures (NA) in illumination and detection. If the wavelengths of illumination and detection are the same, we have ${{\rm{\sigma }}}_{{\rm{il}}}={{\rm{\sigma }}}_{\det }$ and ${\sigma }_{{\rm{ISM}}}=\frac{1}{\sqrt{2}}{{\rm{\sigma }}}_{\det }$. Reduction of the Gaussian PSF width by a $\sqrt{2}$ factor corresponds to the same resolution enhancement in terms of the generalized Rayleigh criterion. But if one of the PSFs, either in detection or illumination, is significantly narrower than the other, then the resolution *σ*_ISM_ of ISM is close to the width of that narrower PSF. This could be the case if the wavelength of detection is greater than that of illumination, e.g., due to the Stokes shift in fluorescence microscopy. For example, if ${\sigma }_{{\rm{il}}}=0.5{\sigma }_{\det }$, then ${\sigma }_{{\rm{ISM}}}\approx 0.89{\sigma }_{{\rm{il}}}$.

For coherent imaging, the amplitude point spread function (APSF) serves as the equivalent of the PSF in the incoherent scenario. It characterizes the field distribution in the image plane generated by a point source in the object plane. The resulting intensity distribution of the image can be calculated as a square of the convolution of the object field with the APSF. For confocal and image scanning cases, the expressions provided above for the incoherent scenario can be adopted by simply replacing PSFs with APSFs (see Table 1)

**Table 1:** Image formation comparison for coherent and incoherent, widefield and image scanning setups

	Widefield	Image scanning
**Incoherent**	${\rm{I}}={\rm{Obj}}\, {\circledast} \,{\rm{PS}}{{\rm{F}}}_{\det }$	${\rm{I}}={\rm{Obj}}\, {\circledast}\, \left({\rm{PS}}{{\rm{F}}}_{\det }\cdot {\rm{PS}}{{\rm{F}}}_{{\rm{il}}}\right)$
**Coherent**	${\rm{I}}=|{\rm{obj}} \,{\circledast}\, {\rm{APS}}{{\rm{F}}}_{\det }{|}^{2}$	${\rm{I}}=|{\rm{obj}} \,{\circledast}\, \left({\rm{APS}}{{\rm{F}}}_{\det }\cdot {\rm{APS}}{{\rm{F}}}_{{\rm{il}}}\right){|}^{2}$

*I* intensity function of the image, *Obj*, *obj* intensity, and amplitude reflections of the object, respectively

It’s noteworthy to acknowledge that while the Gaussian approximation offers a convenient means for estimating resolution improvement, it is not entirely precise. The APSF of a circular aperture is a ${\rm{Jinc}}\left(\cdot \right)$ function (${{\rm{Jinc}}}^{2}\left(\cdot \right)$ for the PSF in the incoherent case). The resolution enhancement according to the generalized Rayleigh criterion for incoherent imaging and identical illumination and detection PSFs, in this case, is about 1.53^17,38^.

### Hermite–Gaussian imaging and scanning

Tsang’s initial proposal to overcome the diffraction limit by examining the HG modes addressed the task of distinguishing two point sources in the far-field and estimating the distance between them^25^. It was shown that if, instead of the standard intensity measurement, one isolates the first-order HG mode and measures its intensity, the Fisher information per photon remains constant even when the sources approach each other. Following this insightful discovery, extensive efforts, both theoretical and experimental, have been invested in exploring broader applications of this technique, extending beyond single-parameter scenarios to encompass imaging tasks. Here we briefly recap the main concepts of HGI from Ref. ^26^.

**Fig. 1:**
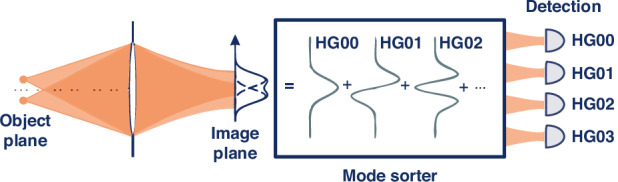
The concept of Hermite–Gaussian imaging via spatial mode demultiplexing. The measurement is carried out by decomposing the optical field in the image plane into the basis of Hermite–Gaussian modes and measuring the amplitudes or intensities for coherent or incoherent imaging, respectively. The outcomes of the measurements approximate geometrical moments of the sample field or intensity distribution in the object plane, and hence permit reconstructing this distribution with a high precision^26^

HGI decomposes the field in the image plane into the Hermite–Gaussian basis, defined as

3 $${\phi }_{{mn}}\left(x,y\right)=\frac{1}{\sqrt{2\pi {\sigma }^{2}}}\frac{1}{\sqrt{{2}^{n+m}n!m!}}{H}_{n}\left(\frac{x}{\sqrt{2}\sigma }\right){H}_{m}\left(\frac{y}{\sqrt{2}\sigma }\right){{\rm{e}}}^{-\frac{{x}^{2}+{y}^{2}}{4{\sigma }_{{\rm{d}}{et}}^{2}}}$$

where ${H}_{n}\left(x\right)$ corresponds to Hermite polynomial of the order *n*. One then measures the amplitude (for coherent imaging) or intensity (for incoherent imaging) of each mode (Fig. 1). These measurements allow one to access the corresponding geometrical moments of the field/intensity distribution of the initial object. For the coherent scenario, one can directly extract the object field distribution from the measured amplitudes. Incoherent HGI measurements yield only even moments of the object intensity distribution, so the HG basis needs to be augmented with HG mode superpositions to access odd moments^27^. Eventually, in both cases, the obtained moments are used to reconstruct the field or intensity of the object^31,35^. The resolution achieved with HGI depends on the number of modes used in the experiment and is limited only by the signal-to-noise ratio as the detectable amount of light in each mode drops exponentially with the mode order. Previous experiments on HGI under widefield illumination have shown resolution improvement by factors of 2–3 compared to direct imaging (DI) both in incoherent and coherent scenarios^26,31,33–35^.

In this work, we implement HGI in the ISM setting by replacing the CCD with HG measurements. At each scanning step, HGI reconstructs a snapshot of the object under restricted illumination with improved resolution. Subsequent pixel reassignment allows us to form a final image of the object based on individual snapshots.

Reconstructing each snapshot from HG measurements is a complex process, in practice requiring a neural network^31^. However, the reconstructed image can be approximated as a convolution of the object with an effective detection (A)PSF. Hence, we can describe the resolution of HGI-based ISM using the same formalism as DI-based ISM as discussed in the previous section.

### Experiment

We use a macroscopic reflective coherent imaging setting on an optical table akin to Refs. ^31,35^. In our proof-of-concept experiment, we aim to maintain complete control over the samples being imaged. To accomplish this, we utilize a digital micromirror device (DMD) to display various objects composed of binary “logical” pixels. Each logical pixel is a square with a side of 10 physical DMD pixels (75.6 μm). The area utilized on the DMD in all experiments is a square of size 210 × 210 physical pixels (hereafter referred to as “frame”). The limitation of this approach is that the features that can be displayed on the DMD are much larger than the wavelength of the laser used for illumination (795 nm). To approach the resolution limit in this case we artificially decrease the numerical aperture (NA) of the imaging system down to $0.71\cdot {10}^{-3}$ by placing an iris in the imaging path at a significant distance (∼2.5 m) away from the object. This iris is the entrance pupil of the imaging system and leads to a direct imaging resolution of ${\sigma }_{{\rm{d}}{et},{DI}}=0.21{\rm{\lambda }}/{\rm{NA}}\simeq 0.23{\rm{mm}}$.

**Fig. 2:**
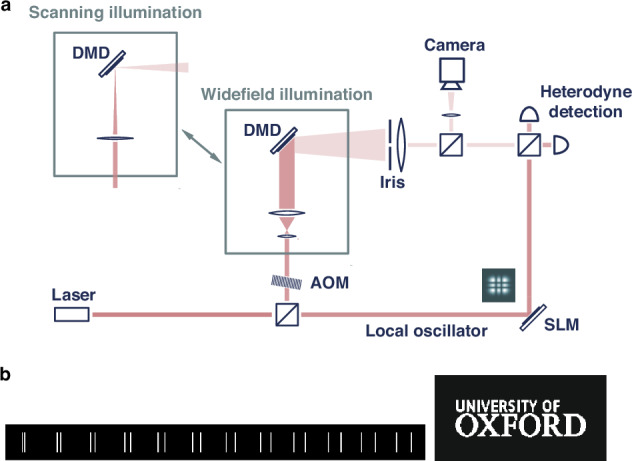
Experiment for comparing DI and HGI under widefield, and focused scanning illumination. **a** Optical setup. The objects in the image are displayed on the DMD. Measurement in HG basis is carried out by a heterodyne detector with the local oscillator sequentially prepared in different HG modes by the SLM. **b** Bitmaps displayed on the DMD for resolution estimation: Oxford University logo and pairs of lines with separation varying from 20 to 130 DMD pixels

We study two objects (Fig. 2b). First, to quantitatively assess the lateral resolution in the experiment, we employ a set of parallel line pairs of 175 DMD pixel lengths with the center-to-center separation varying from 20 to 130 DMD pixels in steps of 10 DMD pixels. Second, we use the Oxford University logo (1680 × 630 DMD pixels) as an example of a more complex object. This object is displayed on the DMD as a sequence of frames as described below.

Unlike typical confocal or ISM setups, the illumination beam and the light reflected from the sample do not share the same optical path in our experiment. This grants us the flexibility to switch between different illumination settings independently of the detection scheme. We compare HGI and DI both with widefield and scanning approaches. Experiments with each of the four combinations are described in the “Materials and methods” section.

**Fig. 3:**
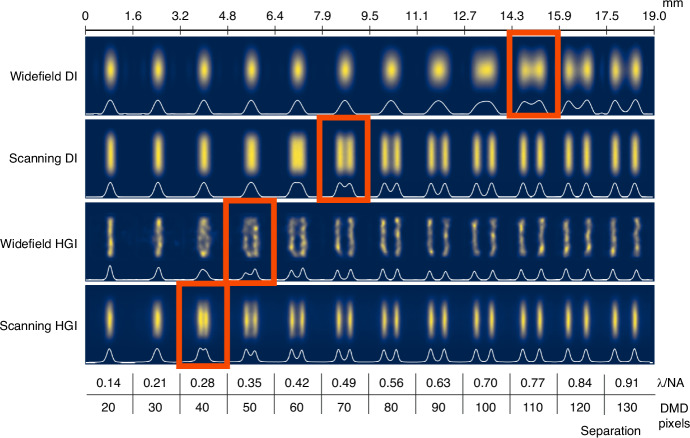
Imaging the pairs of lines with gradually changing separation using DI and HGI under widefield and focused scanning illumination. White lines represent the one-dimensional profile of the line pairs in arbitrary intensity units

To estimate the resolution value for each particular method, we utilize the set of line pairs and interpolate the pixel value to find the distance for the generalized Rayleigh limit (Fig. 3). The estimated resolution for DI is 111 ± 1 DMD pixel being slightly better than the coherent Rayleigh limit estimation of $0.84{\rm{\lambda }}/{\rm{NA}}\simeq 120$ DMD pixels based on the NA measurement. Narrowing the illumination beam and implementing scanning with pixel reassignment enhances the widefield resolution by a factor of 1.6 (69 ± 1 DMD pixels ≃ 0.48 λ/NA) as expected for coherent ISM. The HGI experiment with widefield illumination managed to achieve 46 ± 1 DMD pixel resolution, meaning a factor of 2.4 resolution improvement compared to widefield DI. Finally, HGI with scanning reached 44 ± 1 DMD pixels corresponding to 0.31 λ/NA. The expected resolution improvement of scanning HGI compared to widefield HGI evaluated via Eq. (1) with ${\sigma }_{{\rm{i}}l}/{\sigma }_{{\rm{d}}{et}}=2.4$ cannot exceed 8%, consistent with these observations.

**Fig. 4:**
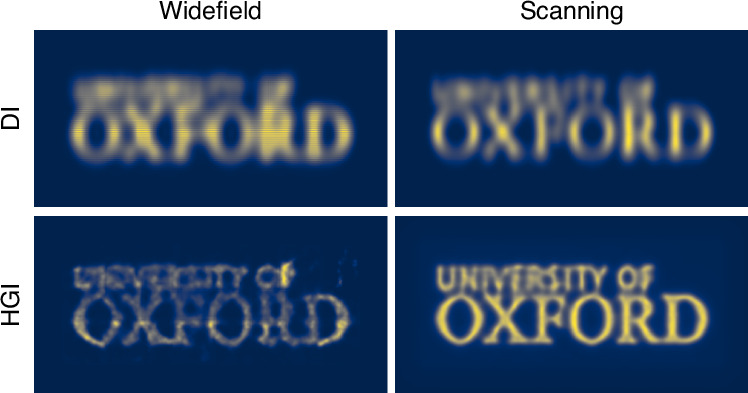
Imaging the Oxford logo using DI and HGI under widefield and focused scanning illumination

The Oxford University logo images produced with different methods (Fig. 4) follow the same trend as the line pairs, with scanning HGI achieving the best quality. Notably, both the line pairs and the logo reveal artifacts in widefield HGI that are distinct from conventional optical blur.

As discussed, the more resolution improvement we get from HGI, the less ISM can add to it. However, as one can observe, the scanning significantly reduces the impact of HGI artifacts on the final image and consequently improves its quality. The reason is that, as mentioned, experimental image formation in HGI can only approximately be described as a convolution with a Gaussian PSF. In practice, the HGI point source response might depend on various factors such as the position in the object plane and the presence of other sources. For example, as seen in Fig. 3, HGI seems to achieve better results in the center of the picture than around the edges. Augmenting HGI with scanning provides translational invariance and averages out the artifacts produced by each individual reconstructed snapshot.

To quantitatively evaluate image quality improvement, we calculate the Multi-Scale Structural Similarity Index Measure (MS-SSIM)^39^. We randomly sample small fragments of the University logo picture reconstructed with each method and compare them with the corresponding region of the ground truth picture (see the corresponding section in the “Materials and methods”). Both the widefield and scanning DI images yield very low MS-SSIM scores below 0.01. While the widefield HGI image shows some improvement with a score of 0.080 ± 0.002 due to the increased resolution, the scanning HGI significantly outperforms it, achieving an MS-SSIM of 0.272 ± 0.006. This result underscores the substantial advantage of scanning HGI over widefield HGI in terms of image quality.

**Fig. 5:**
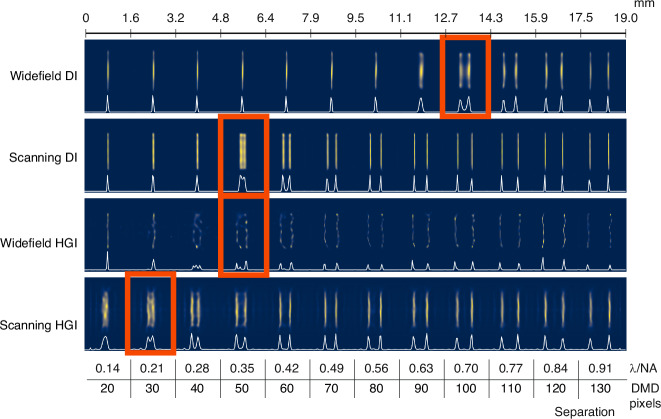
R-L deconvolution applied to the images of line pairs with gradually changing separation obtained via DI and HGI under widefield and focused scanning illumination with R-L deconvolution applied. White lines represent the one-dimensional profile of the line pairs in arbitrary intensity units

**Fig. 6:**
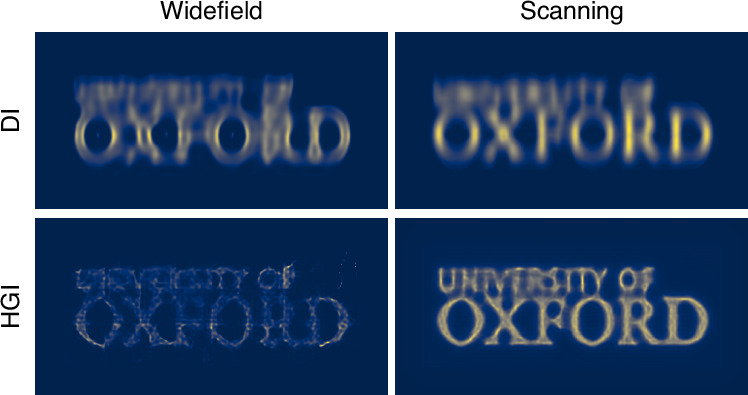
R-L deconvolution applied to the Oxford logo images, obtained via DI and HGI under widefield and focused scanning illumination

When it comes to further processing, ISM is typically followed by a deconvolution step^18^. Since the cut-off spatial frequency in ISM is doubled compared to widefield imaging, this step further enhances the resolution afforded by pixel reassignment, enabling nearly a two-fold improvement compared to widefield imaging. In a similar vein, we apply the Richardson-Lucy (R-L) deconvolution algorithm to all four experimental results, using Gaussian PSFs estimated from the images of the line pairs, with 300 iterations in each case (Figs. 5 and 6). As expected, for coherent DI, the improvement in the line pairs is minimal, while noticeable artifacts appear in the Oxford logo image, consistent with our earlier observations^31^. Scanning DI delivered results consistent with the anticipated two-fold enhancement. Widefield HGI showed no improvement; in fact, it exacerbated the initial artifacts. Finally, scanning HGI benefited significantly from deconvolution, pushing the resolution further to 28 ± 1 DMD pixels (0.20 λ/NA) and achieving almost four-fold enhancement compared to widefield DI.

This factor corresponds to a product of resolution improvement factors of ∼ 2 associated with both techniques (ISM and HGM) taken individually. This is much higher than what is expected according to Eq. (2). The significant additional enhancement provided by deconvolution is a consequence of a much higher quality of an image reconstructed through ISM + HGI (Fig. 4, bottom right) as compared to HGI alone (Fig. 4, bottom left).

## Discussion

To gain a more in-depth understanding of the resolution enhancement observed, let us discuss it in the context of other methods. We assume passive, linear, far-field detection using an objective lens with a cylindrical aperture and allowing illumination engineering. We begin by considering coherent illumination and detection, which is the case in our current experiment. Following Abbe’s theory of resolution, the point spread function APSF(*x*) is the Fourier image of the amplitude transfer function in the objective lens plane ATF(k_⊥_) = $F\left[{\rm{APSF}}\left(x\right)\right]$, which is a top-hat function with the support within |*k*_⊥_| ≤ NA/λ. This determines the resolution limit for coherent imaging, with the sample illuminated with a plane wave perpendicular to the sample.

Illuminating at an angle (oblique illumination) shifts the spatial frequency spectrum of the light reflected from the sample. Assuming that the illuminating light must pass through the same objective lens, this shift cannot exceed NA/λ. Oblique illumination at multiple angles therefore allows access to the range of transverse frequencies within |*k*_⊥_| ≤ 2NA/λ^40^. This translates into doubling the resolution in terms of the Rayleigh limit. The same applies to other engineered illumination methods, including ISM and SIM, as long as both the illumination and imaging are performed through the same objective lens^4,41^.

In incoherent imaging, we detect the intensity, which is the square absolute value of the field amplitude. The intensity point spread function is ${\rm{PSF}}\left(x\right)=|{\rm{APSF}}\left(x\right){|}^{2}$. Its Fourier image — the optical transfer function ${\rm{OTF}}\left({k}_{\perp }\right)={\mathscr{F}}\left[{\rm{PSF}}\left(x\right)\right]$ — is a convolution of the ATF with itself. The OTF has a triangle-like shape with support within 2NA/λ (twice as wide as the corresponding ATF). However, because high-frequency components are reduced compared to low-frequency, the *effective* cut-off frequency defining the resolution associated with this triangular OTF is about 1.5 times lower. Digital processing (deconvolution or inverse filtering) can “amplify” higher frequencies in the final image, making the OTF closer to top-hat, bringing the effective cut-off frequency back to 2NA/λ and thus achieving a resolution that is up to ∼1.5 times higher in terms of the Rayleigh limit than without deconvolution^40^. Similarly to the coherent case, engineering illumination via, e.g., SIM or ISM allows one to enhance the cut-off frequency by an additional factor of 2, i.e., up to 4NA/λ, which corresponds to a factor of ≲3 Rayleigh resolution enhancement compared to undeconvolved imaging with widefield illumination^4,40^.

Deconvolution is however notoriously vulnerable to noise. In direct imaging, weak high-frequency components are detected in a mixture with strong low-frequency components, so the noise of the latter obscures the former. As a result, deconvolution rarely meets its theoretical limits, especially in the case of a limited photon budget and, consequently, significant shot noise. HGI, treating imaging as a quantum sensing problem, optimizes the measurement basis so that this effect is minimized^25,26,30^, thereby allowing one to take full advantage of the information transmitted through the objective lens aperture.

An opportunity to enhance the resolution further opens up if the sample size is limited, as is the case in our experiment. This also applies if only a limited part of the object is illuminated. Mathematically this can be represented by the function ${\rm{Obj}}\left(x\right)$ multiplied by a top-hat “window function” $w\left(x\right)$. The spatial spectrum of this product is then a convolution ${\mathscr{F}}\left[{\rm{Obj}}\left(x\right)w\left(x\right)\right]={\mathscr{F}}[{\rm{Obj}}\left(x\right)]\,{*}\,{\mathscr{F}}\left[w\left(x\right)\right]$. This allows high-frequency components of ${\rm{Obj}}\left(x\right)$ to admix into lower-frequency components of this convolved spectrum and therefore “leak” through the aperture, thereby becoming accessible to measurement^42,43^. These contributions are however extremely weak; they are below the shot noise level assuming any reasonable intensity^40^. Hence they are prohibitively difficult to measure using direct intensity imaging.

One way to address this issue and achieve bandwidth extrapolation is using superoscillation functions either for illumination or as a PSF^44,45^. A shortcoming of this approach is dramatic optical loss due to exponentially large side lobes, which are inevitable for a superoscillating function. HGI instead engineers the measurement to minimize the pollution from low-frequency component noise when detecting the high-frequency components^46^. Applying HGI together with the conventional ISM, we take the best of both worlds: maximize the cut-off frequency of the ATF thanks to structured illumination and perform an optimized measurement of the light transmitted through the objective lens thanks to HGI.

Since HGI incorporates the NN to reconstruct images, it is challenging to disentangle the improvements achieved by the measurement itself from the potential effects of the NN. To isolate the NN’s contribution, we applied a similar NN to the snapshots acquired in the DI experiments. The network failed to produce any noticeable enhancement to the resolution both for widefield and scanning data. We thus conclude that the observed resolution enhancement is due to the new physics of HGI rather than the effects of NN.

We note that heterodyne detection employed in this work is not an optimal way to measure the optical field on an HG basis. Sequential measurements of modes’ amplitudes are inefficient both in terms of the acquisition time and photon budget. This issue can be mitigated by means of digital holography when a complete measurement of the optical field is performed at once. However, a more fundamental obstacle is the shot noise of the LO^47^ which is present both in the heterodyne and holographic methods. The optimal measurement strategy would be to passively decompose the incoming field, directing each HG mode into a separate spatial channel, and then measure each mode individually. Although there has been significant progress in developing optical mode-sorters — devices capable of performing such decomposition^48–50^, the performance of this technology is not yet sufficient for the requirements of our experiment.

For incoherent fluorescent samples, a passive mode-sorter becomes even more critical since LO can no longer be employed due to its limited bandwidth. Notably, HGI offers even greater resolution enhancement for incoherent objects^27,28,35^, and we anticipate that it would similarly benefit from the scanning approach.

In summary, in this study, we introduced a novel approach by integrating the HGI technique with the conventional ISM superresolution method. We experimentally demonstrate how both methods benefit from complementing each other. Although in the HGI setting the additional resolution enhancement due to ISM was limited for the raw images, we observed substantial improvement in the overall image quality, which in turn allowed us to successfully employ the R-L deconvolution algorithm. The ultimate resolution improvement for coherent imaging in our experiment reached a factor of 2.5 and further increased to a factor of 4 by deconvolution. This is, to the best of our knowledge, the first combination of resolution enhancement methods applied at the illumination and detection ends of the imaging device with a cumulative effect. It is likely that HGI can be combined similarly with other resolution enhancement methods based on structuring the illumination pattern. The ultimate resolution improvement that can be potentially obtained with scanning HGI taking into account experimental caveats and digital processing remains an open question.

This result makes the combined strategy promising for imaging of real-world specimens. The next important step is to apply the proposed method to imaging of microscopic samples through high NA objectives. A major hurdle here lies in training the NN for HGI snapshot reconstruction as supervised learning requires a ground truth knowledge of the training set. Another important milestone is the development of an efficient passive mode-sorter that will allow imaging of incoherent fluorescent specimens.

## Materials and methods

### Image scanning microscopy

Let us consider the incoherent case. We assume the illumination beam stays centered in the object plane, so its intensity distribution is (${\rm{PS}}{{\rm{F}}}_{{\rm{il}}}\left(x\right)$), and the sample, whose spatial distribution is denoted by Obj(⋅) is displaced by *S*. The resulting intensity distribution in the image plane can be expressed as

4 $$I\left({y|S}\right)=\int {\rm{Obj}}\left(x+S\right)\cdot {\rm{PS}}{{\rm{F}}}_{{\rm{il}}}\left(x\right)\cdot {\rm{PS}}{{\rm{F}}}_{\det }\left(y-x\right){dx}$$

Substituting the illumination and detection PSF profiles we obtain

5 $$\begin{array}{c}{\rm{PS}}{{\rm{F}}}_{{\rm{il}}}\left(x\right)\cdot {\rm{PS}}{{\rm{F}}}_{\det }\left(y-x\right)\propto \exp \left(-\frac{{x}^{2}}{2{\sigma }_{{\rm{il}}}^{2}}\right)\cdot \exp \left(-\frac{{\left(x-y\right)}^{2}}{2{\sigma }_{\det }^{2}}\right)\propto \\ \propto \exp \left(-\frac{{y}^{2}}{2{\sigma }_{\det }^{2}}\right)\cdot \exp \left(-\frac{{\left(x-\frac{{\sigma }_{{\rm{ISM}}}^{2}}{{\sigma }_{\det }^{2}}y\right)}^{2}}{2{\sigma }_{{\rm{ISM}}}^{2}}\right)\end{array}$$

where

6 $${\sigma }_{\rm{ISM}}=\frac{{\sigma }_{{il}}}{\sqrt{{\sigma }_{{il}}^{2}+{\sigma }_{\det }^{2}}}\cdot {\sigma }_{\det }$$

and we used the proportionality sign to contain all constants (independent of *x*, *y*, and *S*). Hence

7 $$I\left({y|S}\right)\propto \exp \left(-\frac{{y}^{2}}{2{\sigma }_{\det }^{2}}\right)\int {\rm{Obj}}\left(x+S\right)\cdot \exp \left(-\frac{{\left(x-\frac{{\sigma }_{{\rm{ISM}}}^{2}}{{\sigma }_{\det }^{2}}y\right)}^{2}}{2{\sigma }_{{\rm{ISM}}}^{2}}\right){dx}$$

Let us rescale the acquired single-shot image by defining a new function:

8 $$I^{\prime} \left({y|S}\right)=I\left(\frac{{\sigma }_{\det }^{2}}{{\sigma }_{{\rm{ISM}}}^{2}}{y|S}\right)$$

This rescaled image is a convolution of the object at the current position with a narrower Gaussian PSF. The result of the convolution is also multiplied by a decaying exponent meaning that the image is only produced for the illuminated part of the object:

9 $$I^{\prime} \left({y|S}\right)\propto \exp \left(-\frac{{y}^{2}}{2{\sigma }_{\det }^{2}}\right)\int {\rm{Obj}}\left(x+S\right)\cdot \exp \left(-\frac{{\left(x-y\right)}^{2}}{2{\sigma }_{{\rm{ISM}}}^{2}}\right){dx}$$

We can replace the integration variable for $x^{\prime} =x+S$:

10 $${I^{\prime} \left({y|S}\right)\propto \exp \left(-\frac{{y}^{2}}{2{\sigma }_{\det }^{2}}\right)\int {\rm{Obj}}\left(x^{\prime} \right)\cdot \exp \left(-\frac{{\left(x^{\prime} -y-S\right)}^{2}}{2{\sigma }_{{\rm{ISM}}}^{2}}\right){dx},}$$

To obtain the full image, we sum all the single-shot images, displaced by the corresponding shift *S*:

11 $${I}_{{\rm{I}}{SM}}\left(y\right)=\int I^{\prime} (y-{S|S}){dS}\propto \iint \exp \left(-\frac{{\left(y-S\right)}^{2}}{2{\sigma }_{\det }^{2}}\right){\rm{Obj}}\left({x}^{{\prime} }\right)\exp \left(-\frac{{\left({x}^{{\prime} }-y\right)}^{2}}{2{\sigma }_{{\rm{ISM}}}^{2}}\right)d{x}^{{\prime} }{dS}$$

We notice that the double integral can be separated:

12 $${I}_{{\rm{I}}{SM}}\left(y\right)\propto \int \exp \left(-\frac{{\left(y-S\right)}^{2}}{2{\sigma }_{\det }^{2}}\right){dS}\cdot \int {\rm{Obj}}\left(x^{\prime} \right)\cdot \exp \left(-\frac{{\left(x^{\prime} -y\right)}^{2}}{2{\sigma }_{{\rm{ISM}}}^{2}}\right){{dx}}$$

The first integral is a Gaussian integral: its value is constant and can be absorbed into the proportionality sign:

13 $${{I}_{{\rm{ISM}}}\left(y^{\prime} \right)=\sum _{i}{I}^{{\prime} }({y}^{{\prime} }|{S}_{i})\propto \int {\rm{Obj}}\left(x^{\prime} \right)\cdot \exp \left(-\frac{{\left(x^{\prime} -y^{\prime} \right)}^{2}}{2{\sigma }_{{\rm{ISM}}}^{2}}\right){dx}}$$

We see that the result of this procedure, known as pixel reassignment, is a convolution of the object intensity distribution with ${\rm{PS}}{{\rm{F}}}_{{\rm{ISM}}}\left(\cdot \right)$.

### Experimental setup

#### Widefield DI

DI is performed by imaging the DMD onto the CCD camera with a set of lenses. The collimated illumination beam is larger than the DMD screen and can be considered uniform within the displayed frame region. For the line separation measurement, each pair is displayed and acquired separately. To image the university logo, which is larger than the DMD frame size, we cut it into a number of overlapping frames and displayed them one by one. After collecting the images, we crop out the central part before stitching them together. Removing the edges in each picture minimizes the impact of optical aberrations on the image quality and allows for a fairer comparison of the methods purely from the resolution perspective^31^.

#### Scanning DI

Our DI scanning experiment mimics the ISM setting on a macroscopic scale. We focus the illumination beam so that the waist of ${\rm{APS}}{{\rm{F}}}_{{\rm{il}}}$ on the DMD is significantly less than the displayed frame size. To emulate the real-world scenario when ${\rm{APS}}{{\rm{F}}}_{{\rm{il}}}$ and ${\rm{APS}}{{\rm{F}}}_{\det }$ are equal, we independently measure the corresponding intensity distributions and match them. The measured *σ*-parameter of ${\left|{\rm{APS}}{{\rm{F}}}_{\det }\right|}^{2}$ and ${\left|{\rm{APS}}{{\rm{F}}}_{{\rm{il}}}\right|}^{2}$ is 28 DMD pixels (212 μm) and is slightly less than the value estimated from NA (30 DMD pixels).

Once the illumination is set in the center of the DMD frame, it stays constant and scanning is emulated by displaying displaced objects on the DMD. At each step, the pattern on the DMD is shifted by 20 DMD pixels and the snapshot is captured by the camera. After collecting all individual snapshots we recombine them using the pixel reassignment routine to get an improved-resolution image of the initial object.

#### Widefield HGI

For widefield HGI we reproduce the coherent imaging experiment described in the previous work^31^. Measurement in the HG basis is carried out by means of heterodyne detection, with the local oscillator (LO) sequentially prepared in each of the 441 HG modes of orders 0 to 20 in both transverse dimensions.

The light from the laser is split into two paths. One of them is used to illuminate the sample whilst the other serves as the LO. An acousto-optic modulator (AOM) introduces a frequency shift of 80 MHz to the illumination beam and a spatial light modulator (SLM) is utilized for the LO mode preparation. After reflecting from the DMD and passing through the iris, the optical signal is recombined with the LO mode on a beamsplitter, and the output is measured with a balanced detector to extract the amplitudes and phases corresponding to each HG mode for every individual sample.

To compensate for various imperfections of the optical setup, we use a fully connected neural network (NN) with four hidden layers that transform HG measurements into images. The training set comprises 2 × 10^4^ patterns of random patterns, lines, and ellipses displayed on the DMD. The corresponding photocurrents measured for different HG modes are the inputs of the NN. The labels are the ground truth images that are slightly smeared to avoid overfitting^31^. Once trained, the NN is utilized to reconstruct the objects of interest that were not part of the training set.

#### Scanning HGI

The optical setup for scanning HGI is modified in a similar manner as for the scanning DI counterpart by narrowing the light beam illuminating the DMD. The NN training set is the same as for the widefield HGI, however, for label generation we take into account the narrow illumination of the samples, so the label images fade to black outside the illuminated region.

Once the NN is trained, the test set of objects is imaged. Each object is displayed with multiple shifts along both axes to implement scanning. For each step, the snapshot is reconstructed using the NN. Subsequently, individual snapshots are combined through pixel reassignment to derive the final image.

### Neural network for HGI image reconstruction

Imperfections in the experimental setup hinder direct image reconstruction from the measured HG mode amplitudes. To overcome this challenge we employ a NN that is trained to perform the reconstruction^31,35^.

In this work, we use a fully connected network with four hidden layers, comprising 800, 400, 400, and 800 hidden neurons, respectively. The activation functions are hyperbolic tangent (tanh) in the first hidden layer, while the remaining three layers use rectified linear units (ReLU). To ensure non-negative image output, the final layer uses a sigmoid activation function. The input layer consists of 882 neurons, representing the real and imaginary parts of the measured field amplitudes from a 21 by 21 set of HG modes. The output layer contains 2500 neurons, corresponding to a 50 by 50 pixel image. This architecture, which is similar to the one employed in Ref. ^35^ matches the performance of the 2-layer fully connected NN with 6000 hidden neurons per layer used in Ref. ^31^, but significantly reduces the training time — taking less than an hour compared to several hours. Adding more layers or neurons to the current architecture did not further improve training accuracy.

The training dataset is generated in the same way as in Refs. ^31,35^, consisting of 10,000 random logical pixel bitmaps with 20–80% fill factor, 5000 random ellipses, and 5000 random straight lines of variable width. This dataset is randomly divided into the training and cross-validation subsets with a 9:1 ratio.

**Fig. 7:**
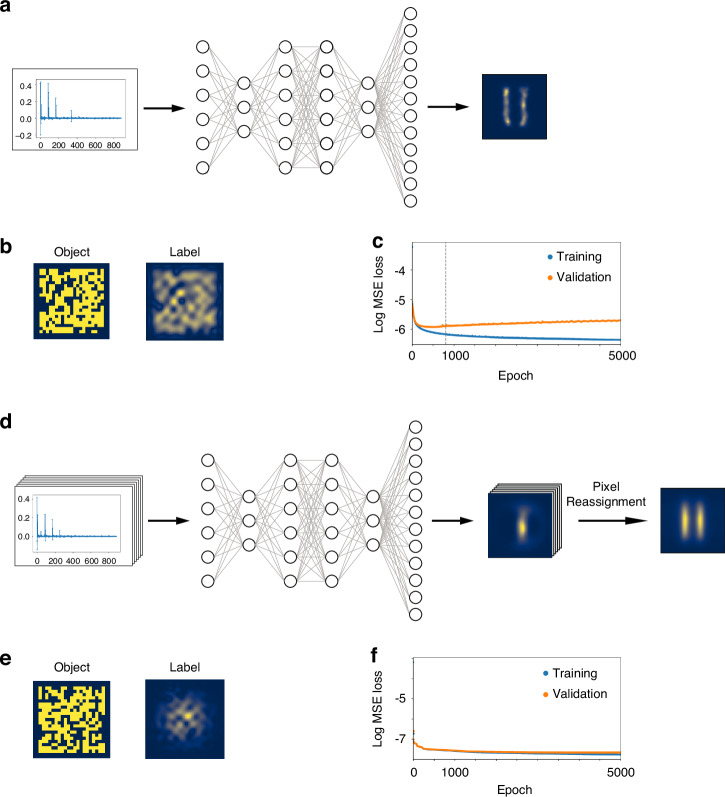
Fully connected NN applied to the HGI reconstruction. **a** Reconstruction of an image from experimentally measured photocurrents for HGI widefield; **b** example of an object from the training set that is displayed on the DMD and the label used for the NN training of widefield HGI; **c** training and cross-validation loss curves for widefield HGI; **d** reconstruction of an image from experimentally measured photocurrents for scanning HGI, the NN step is followed by pixel reassignment; **e** example of an object from the training set that is displayed on the DMD and the label used for the NN training, the label is constructed with focused illumination taken into account; **f** training and cross-validation loss curves for scanning HGI

The training labels are produced from the original objects of the training set by simulating perfect HGI reconstruction with the same set of modes as is used in the experiment. The labels for the widefield and scanning data are different as shown in Fig. 7b, e, taking into account widefield *vs* focused illumination of the object.

The training is conducted with a batch size of 32, using mean squared error (MSE) as the loss function. The Adam optimizer is employed with exponential moving average parameters of 0.9 for the first moment and 0.999 for the second one and no weight decay. The learning rate is set to 10^−4^. The hardware utilized for training is an 11th Generation Intel Core i9-11900K CPU, 64 GB of memory, and an NVIDIA RTX A5000 GPU with 24 GB of memory. For both widefield and scanning data, the training is carried out for 5000 epochs, however, widefield shows signs of overfitting (Fig. 7c) quite early so we pick the weights from the 800th epoch as they show the best performance. Interestingly, for the scanning data overfitting is not observed (Fig. 7f) so we use the weights after 5000 training epochs.

**Fig. 8:**
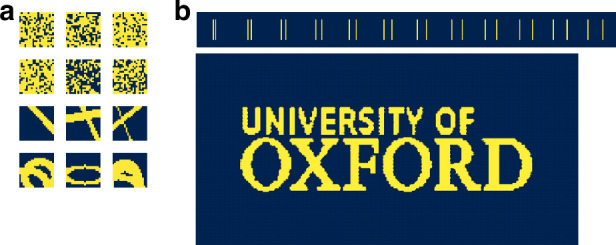
Bitmaps displayed on the DMD. **a** Examples of the objects from the training set. **b** Objects of the test set

Once the training is completed, the NN is applied to the test objects: a set of line pairs and the Oxford University logo (Fig. 2b) which are not part of the training set. For widefield HGI, the test set consists of 36 objects — 21 line pairs (only 12 of them with separation between 20 and 130 DMD pixels, are shown, as the difference between the methods’ performance lies in this interval) and 24 (8 by 3) parts of the Oxford University logo. For scanning HGI the test set is significantly larger as an image on each scanning step needs to be reconstructed: the total number of objects for the line pairs is 1896 (316 by 6) and 4085 (95 by 43) for the Oxford University logo (Fig. 8). The final image is produced by applying pixel reassignment to the reconstructed snapshots.

### MS-SSIM for image quality assessment

**Fig. 9:**
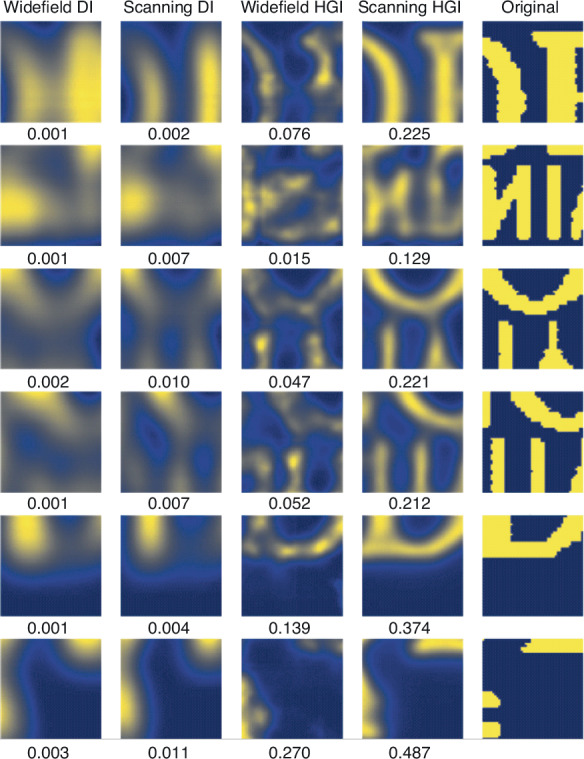
Examples of the samples from the University logo evaluated to calculate MS-SSIM. For each image, the calculated MS-SSIM score is mentioned below

**Fig. 10:**
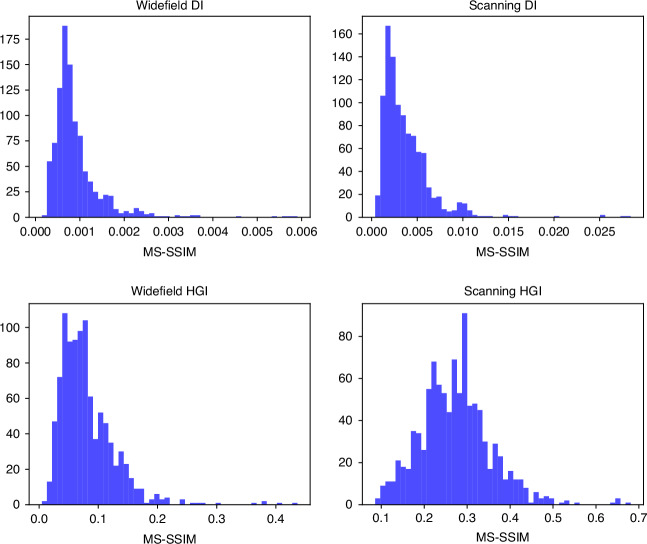
MS-SSIM statistics over the sampled set

To evaluate the MS-SSIM score on the test set, we randomly sample 1000 images 50 by 50 pixels from the large University logo image. Before sampling is performed, the middle part (415 by 125 pixels) of the logo is cropped out to avoid comparing empty sub-images on the borders. The examples of the sampled pictures and the histograms of the MS-SSIM distributions for all four methods are shown in Figs. 9 and 10.

## Data Availability

Data underlying the results presented in this paper are not publicly available at this time but may be obtained from the authors upon reasonable request.
